# Occurrence of β-lactamases among Enterobacterales isolates from 22 US hospitals in a 10-year period: report from the International Network for Optimal Resistance Monitoring (INFORM) programme

**DOI:** 10.1093/jacamr/dlag142

**Published:** 2026-07-24

**Authors:** Mariana Castanheira, Timothy B Doyle, Lalitagauri M Deshpande, Helio S Sader

**Affiliations:** Element Iowa City (JMI Laboratories), 345 Beaver Kreek Centre, Suite A, North Liberty, IA 52317, USA; Element Iowa City (JMI Laboratories), 345 Beaver Kreek Centre, Suite A, North Liberty, IA 52317, USA; Element Iowa City (JMI Laboratories), 345 Beaver Kreek Centre, Suite A, North Liberty, IA 52317, USA; Element Iowa City (JMI Laboratories), 345 Beaver Kreek Centre, Suite A, North Liberty, IA 52317, USA

## Abstract

**Background:**

We analysed 10 years of data from the INFORM programme, which surveys β-lactamases and the activity of ceftazidime-avibactam in US hospitals.

**Methods:**

A total of 33 701 Enterobacterales isolates consecutively collected in 22 US hospitals during 2013–22 were susceptibility tested. *Escherichia coli* and *Klebsiella pneumoniae* displaying MICs ≥ 2 mg/L for two of ceftriaxone, ceftazidime, cefepime or aztreonam; or carbapenem-resistant Enterobacterales (CRE) were evaluated for the presence of β-lactamase genes using microarray/PCR (2013–15) or whole genome sequencing (2016–22).

**Results:**

ESBLs were detected among 2325/2681 (15.5% of 14 923 overall non-CRE isolates) *E. coli* and 874/954 (13.3% of 6572) *K. pneumoniae*. The most common ESBL in both groups was CTX-M-15–like (10.4% of *E. coli* and 10.7% of *K. pneumoniae* isolates). A decline in *E. coli* producing CTX-M-15–like was noted in 2022 (12.6% in 2021 and 8.3% in 2022), and SHV ESBLs had a steady decline among *K. pneumoniae* since 2015. CRE isolates declined steadily from 1.6% in 2013 to 0.8% in 2022. Carbapenemase-positive isolates also declined from 1.3% to 0.5%, with a decline in KPC producers but an increase in isolates producing NDM; NDM producers were initially detected in 2015 and ranged from 1 to 7 isolates collected in the last 5 study years. Ceftazidime-avibactam and the carbapenems are the most active agents against β-lactamase–producing isolates.

**Conclusions:**

Changes in β-lactamase–producing isolates and their susceptibility profiles should be closely monitored at a local and global level since these shifts impact patient treatment, but longitudinal data such as this information is scarce.

## Introduction

Despite the growing threat posed by β-lactamase–producing pathogens, large-scale longitudinal surveillance of Enterobacterales isolates harbouring these enzymes remains limited. Several challenges hinder the implementation of such studies, including complex logistics, the high cost to perform genetic identification of β-lactamase genes and the need for centralized testing to ensure data homogeneity.^1,2^

The International Network for Optimal Resistance Monitoring (INFORM) programme has been monitoring β-lactamase–encoding genes and the *in vitro* activity of ceftazidime-avibactam and comparator agents in US hospitals since 2012.^3^ In this study, we analysed 10 years of data (2013–22) to report trends in β-lactamase production and the susceptibility profiles of isolates carrying these enzymes that were collected from 22 US hospitals throughout the study period.

## Materials and methods

### Bacterial isolates

A total of 33 701 Enterobacterales isolates were consecutively collected in 22 US hospitals participating in the INFORM programme every year from 2013 to 2022.^4,5^ One isolate per patient episode was collected according to a standardized protocol requesting consecutive isolates recovered during January to October of each year from hospitalized patients with pneumonia (100–120 isolates), bloodstream infections (60–100 isolates; approximately 10/month), urinary tract infections (50–60 isolates), intra-abdominal infections (30 isolates) and skin and skin structure infections (100 isolates) that were considered the significant cause of infection according to local criteria. Each hospital could choose the collection period within this timeframe, provided that isolates were collected consecutively. Isolates included in this study were collected from bloodstream infections (1238 isolates; 26.4% overall), skin and skin structure infections (514 isolates; 10.9% overall), pneumonia in hospitalized patients (1267 isolates; 27.0% overall), urinary tract infections (1251 isolates; 26.6% overall) and intra-abdominal infections (427 isolates; 9.1% overall). Organism identifications were confirmed as needed using Matrix-Assisted Laser Desorption Time-of-Flight Mass Spectrometry (MALDI-TOF MS; Bruker, Billerica, Massachusetts).

### Antimicrobial susceptibility testing

Susceptibility testing was performed using the reference broth microdilution method as described by the Clinical and Laboratory Standards Institute (CLSI) M07 (2022) and M100 (2025)^6,7^ documents.

Quality control (QC) was performed according to the CLSI M100 (2025) criteria, and all QC MIC results were within acceptable ranges. Categorical interpretations for all comparator agents were those criteria found in the CLSI M100 (2025)^8^ or the US Food and Drug Administration website.^9^

### Detection of β-lactamases

*Escherichia coli* and *Klebsiella pneumoniae* isolates displaying MIC values ≥ 2 mg/L for ≥2 of the following agents, ceftazidime, ceftriaxone, aztreonam or cefepime, were submitted to genetic characterization. Additionally, all Enterobacterales displaying meropenem and/or imipenem MIC results at >1 mg/L were selected for further analysis. Isolates collected from 2013 to 2015 were screened using a microarray-based assay (Check-MDR CT101 kit; Check-points; Wageningen, Netherlands),^10^ and isolates collected from 2016 to 2023 were submitted to whole genome sequencing and *in silico* analysis as previously described.^11^

## Results

### ESBLs and transferrable cephalosporinases among *E. coli* and *K. pneumoniae*

ESBL-encoding genes were detected among 2325/2682 *E. coli* isolates displaying elevated MIC values for extended-spectrum cephalosporins without carbapenem resistance isolates, corresponding to 86.7% of the isolates screened and 15.5% of the overall *E. coli* isolates collected (*n* = 14 923). Similarly, an ESBL-encoding gene was noted among 874/954 *K. pneumoniae* isolates corresponding to 91.6% of the isolates screened and 13.3% of the overall isolates (*n* = 6572).

Genes encoding CTX-M variants closely related to *bla*_CTX-M-15_ were the most common ESBL-encoding genes detected. CTX-M-15–like encoding genes were detected among 58.3% (1563/2682) of the screened *E. coli* (10.4% overall isolates from this species) and 73.4% (700/954) of the *K. pneumoniae* [10.7% overall; Table S1 (available as Supplementary data at *JAC-AMR* Online)]. When analysing 2072 *E. coli* and 745 *K. pneumoniae* isolates recovered from 2016 to 2022 that have all been submitted to whole genome sequencing, CTX-M-15 was observed among 1084 (91.5% of the ESBL-producers) *E. coli* and 549 (73.7%) *K. pneumoniae* isolates.

CTX-M-9–like was the second most common ESBL detected among *E. coli* isolates. A total of 727 isolates collected in the entire study period harboured variants of CTX-M-9. Among ESBL-producing isolates submitted to sequencing from 2016 to 2022, CTX-M-27 was the most common variant (423/2071 isolates) followed by CTX-M-14 (141 isolates). Only 38 *K. pneumoniae* isolates carried CTX-M-9–like. In this species, the second most common ESBL detected were SHVs with an extended spectrum. This ESBL was detected in 169 isolates (17.7% of qualified isolates and 2.6% overall). Sequencing of the 2016–22 isolates showed that 18 different SHV ESBL variants were detected, the most common being SHV-12 (134 isolates) followed by SHV-7 (44 isolates).

Other ESBL-encoding genes were uncommon for both species, but the gene encoding OXA-1 was detected among 648 *E. coli* and 369 *K. pneumoniae* isolates collected from 2016 to 2022. Among these 1017 isolates submitted to whole genome sequencing, OXA-1 was detected in isolates carrying CTX-M-15–like genes in 631 *E. coli* and 361 *K. pneumoniae*.

Transferable AmpC genes were noted among 267 (10.0% screened and 1.8% overall) *E. coli* and only 32 (3.4% screened and 0.5% overall) *K. pneumoniae*. The genes encoding CMY-2 (139 isolates) and DHA-1 (37) were the most common among isolates submitted to whole genome sequencing after 2015.

**Figure 1.**
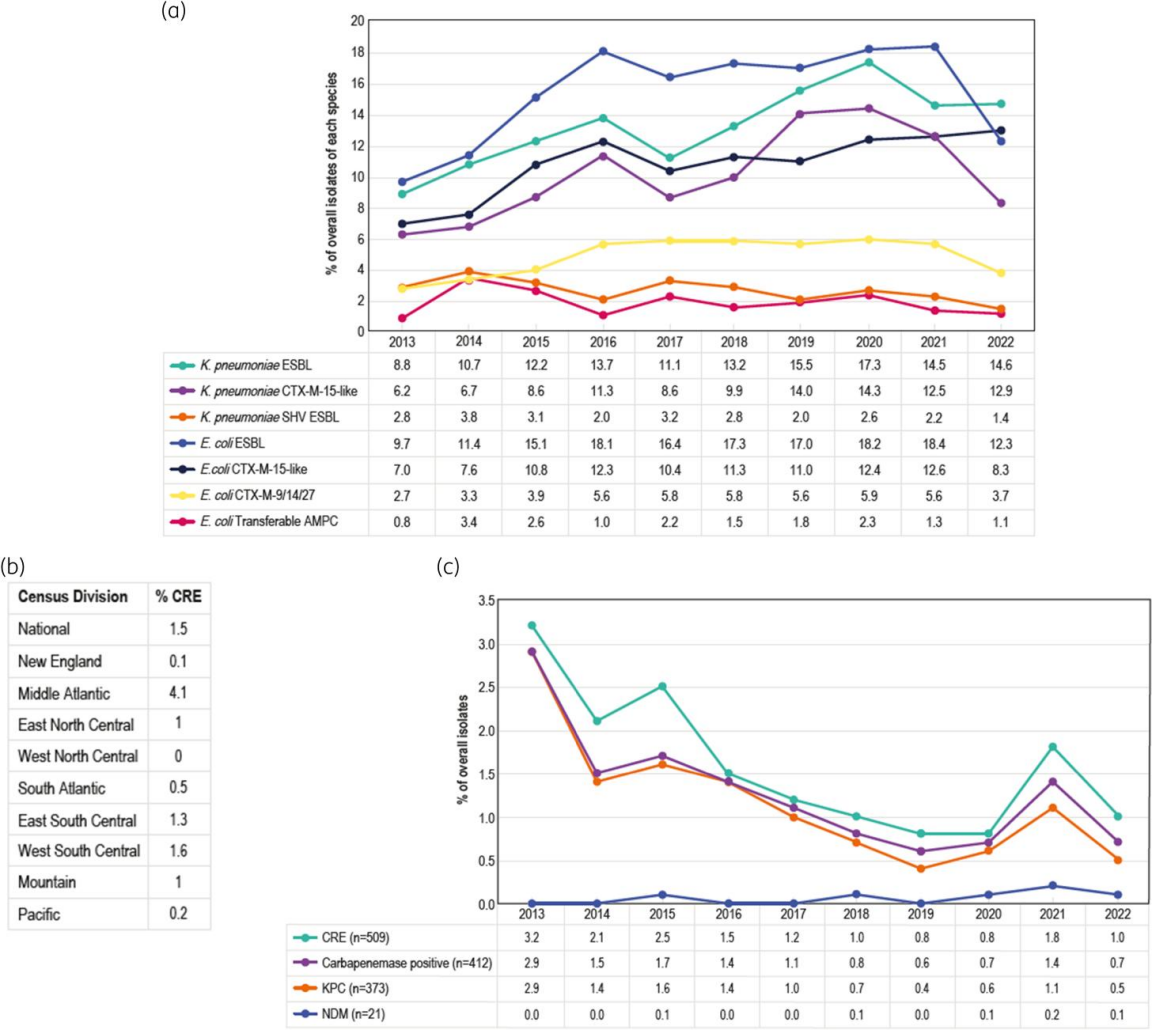
(a) Rates of ESBLs and transferable AmpC among *E. coli* and *K. pneumoniae*. (b) Rates of CRE by US Census Divisions (c) and by enzyme type over 10 years.

*E. coli* isolates carrying an ESBL-encoding gene producing CTX-M-15–like or CTX-M-9/14/27 significantly increased until 2021, but a slight decline in these rates was noted in 2022 (Figure 1a). Transferable AmpC enzymes among *E. coli* increased in 2014 and 2015 but slightly declined since then.

Similar to the trends observed in *E. coli* isolates, ESBL- and CTX-M-15–producing *K. pneumoniae* significantly increased during the study despite a slight decline in 2021–22. On the other hand, SHV ESBLs among *K. pneumoniae* had a steady decline since 2017; SHV ESBL rates were 3.8% in 2015 and declined to 1.4% in 2022 (Figure 1a).

**Figure 2.**
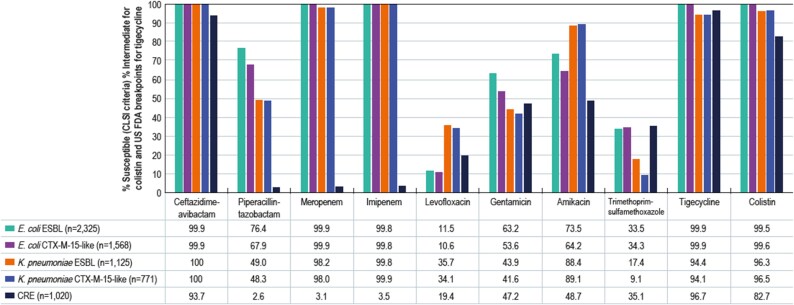
Activity of antimicrobial agents tested against β-lactamase–producing isolates collected during 10 years in 22 US hospitals.

The carbapenems and ceftazidime-avibactam were the most active agents against isolates producing ESBLs or CTX-M-15–like (Figure 2). Piperacillin-tazobactam inhibited 48.7%–76.4% of the ESBL- and CTX-M-15–like producing isolates, with susceptibility rates much higher for *E. coli* (76.4% of the ESBL) when compared to *K. pneumoniae* subsets (49.0%).

### Carbapenemases among Enterobacterales

CREs represented 1.5% (509/33 701) of Enterobacterales isolates (Figure 1b). These isolates were 310 *K. pneumoniae*, 63 *Enterobacter cloacae* species complex, 36 *Serratia marcescens*, 34 *E. coli*, 26 *Klebsiella oxytoca*, 20 *Klebsiella aerogenes*, 10 *Citrobacter freundii* species complex, 7 *Raoultella* spp. and one each of *Hafnia alvei*, *Proteus mirabilis* and *Providencia rettgeri*. The rates of CRE isolates varied in US Census Divisions (Figure 1b).

Carbapenemase-encoding genes were detected among 412 isolates, 1.2% overall and 80.9% of the CRE isolates. These genes included 370 *bla*_KPC_, 21 *bla*_NDM_, 8 *bla*_SME_-like, 7 *bla*_OXA-48_–like, 2 *bla*_VIM-1_ and 2 *bla*_IMP-4_ (Table S2). Among 285 CRE isolates collected from 2016 to 2022 that were submitted to whole genome sequencing, genes encoding KPC-3 were most common followed by KPC-2.

CRE isolates collected declined steadily from 3.2% in 2013 to 1.0% in 2022 (Figure 1c). Carbapenemase-positive isolates also declined from 2.9% to 0.7%, with a decline in KPC producers (2.9% in 2013 to 0.5% in 2022) but an increase in isolates producing NDM. NDM-producing isolates were initially detected in 2015 and have ranged from 2 to 7 isolates each of the last 3 study years.

Against CREs, ceftazidime-avibactam (93.7% susceptible) and tigecycline (96.7% susceptible) were the most active agents (Figure 2). Amikacin and gentamicin inhibited 48.7% and 47.2% of the CRE isolates and 82.7% of the isolates were intermediate to colistin applying the CLSI breakpoints. The susceptibility rates overtime for these antimicrobial agents tested against the main organism groups are displayed in Figure S1.

## Discussion

This study documents the occurrence of ESBL and transferrable cephalosporinases among *E. coli* and *K. pneumoniae* and carbapenemases among Enterobacterales isolates collected in 22 US medical centres during a 10-year period. An overall decrease in β-lactamase–producing isolates was observed in 2022 when compared to prior years, but ESBL genes were still prevalent in US hospitals.

CRE rates were declined during the study period and that was mainly associated with a decrease in KPC-producing isolates. An increase in NDM producers was noted over the years. CREs isolates displayed high resistance rates to aminoglycosides, fluoroquinolones and trimethoprim-sulfamethoxazole. Ceftazidime-avibactam and tigecycline were the only agents tested in this 10-year period displaying activity against CRE isolates.

Despite the reliable activity of ceftazidime-avibactam, this combination agent is not active against isolates producing MBLs, such as NDM enzymes. The increase of NDM-producing isolates in US hospitals highlights the need to not only know if the organism is a CRE but also what type of enzyme it produces to guide appropriate therapy. A recent report from the Antimicrobial Resistance Laboratory Network of the CDC emphasized that treatment should be tailored to the carbapenemase type.^12^

This study highlights the importance of monitoring changes in the prevalence of β-lactamase–producing isolates and their susceptibility profiles at a local and global level since both impact patient treatment. A study by Tamma *et al*. concluded that patients with bloodstream infections caused by ceftriaxone-resistant *E. coli* that can be used as surrogate for ESBL production were more likely to have prolonged hospital stays, to remain in the hospital at Day 30 and to be newly transferred to long-term care facilities.^13^ Similar evidence has been published about serious infections caused by CREs. CRE infections have been associated with longer hospital stays and increased hospitalization costs in different patient populations despite the lack of evidence of increased mortality.^14–16^ Collectively, these observations highlight the continuous need for surveillance, stewardship and use of diagnostics to manage the treatment of β-lactamase–producing Enterobacterales isolates, prevent the spread of resistant strains and mitigate the clinical and economic burden associated with infections caused by these organisms.

## Supplementary Material

dlag142_Supplementary_Data

## Transparency declarations

Element Iowa City (JMI Laboratories) was contracted to perform services in 2022–24 for AimMax Therapeutics, Amicrobe, Inc., Appili Therapeutics, Armata Pharmaceuticals, Astellas Pharma, Inc., Basilea Pharmaceutica AG, Biosergen AB, Bugworks, Cerba Research NV, Cidara Therapeutics, Cipla USA Inc., ContraFect Corporation, CorMedix Inc., Crestone, Inc., Curza Global, LLC, Diamond V, Discuva Ltd, Entasis Therapeutics, Enveda Biosciences, Evopoint Biosciences, Fedora Pharmaceuticals, Fox Chase Chemical Diversity Center, Genentech, Gilead Sciences, Inc., GSK plc, Iterum Therapeutics plc, Janssen Biopharma, Johnson & Johnson, Kaleido Biosciences, LifeMine Therapeutics, Medpace, Inc., Lysovant Sciences, Inc., Meiji Seika Pharma, Melinta Therapeutics, Menarini Group, Merck & Co., MicuRx Pharmaceutical Inc., Mundipharma International Ltd, Mutabilis, Nabriva Therapeutics, National Cancer Institute, National Institutes of Health, Ohio State University, Omnix Medical Ltd, Paratek Pharmaceuticals, Pfizer, PolyPid Ltd, PPD, Prokaryotics, Inc., Pulmocide Ltd, Qpex Biopharma, Revagenix, Roche Holding AG, Roivant Sciences, Scynexis, Inc., Shionogi & Co., Ltd, Sinovent Pharmaceuticals, Inc., Spero Therapeutics, Sumitovant Biopharma, Inc., TenNor Therapeutics, US Food and Drug Administration, VenatoRx Pharmaceuticals, Washington University, Watershed Medical, LLC, Wockhardt and Zoetis, Inc.

## Supplementary data

Figure S1 and Tables S1 and S2 are available as Supplementary data at *JAC-AMR* Online.
