# Supplementary material for: Occurrence of β-lactamases among Enterobacterales isolates from 22 US hospitals in a 10-year period: report from the International Network for Optimal Resistance Monitoring (INFORM) programme
Source: JAC Antimicrob Resist. 2026 Jul 24;8(4):dlag142. doi: 10.1093/jacamr/dlag142 (PMC13397114; doi:10.1093/jacamr/dlag142)

Supplementary Table 1. Counts of *E*. *coli* and *K. pneumoniae* isolates displaying elevated MIC values for extended-spectrum cephalosporins without carbapenem resistance

| **Organism/Year** | **No. of isolates** | | | | | |
| --- | --- | --- | --- | --- | --- | --- |
|  | **Total** | **ESBL gene positive** | **CTX-M-15-like positive** | **CTX-M-9-like positive** | **SHV ESBL positive** | **Transferrable AmpC positive** |
| *E. coli* |  |  |  |  |  |  |
| 2013 | 1305 | 127 | 92 | 35 | 1 | 10 |
| 2014 | 1258 | 144 | 96 | 41 | 5 | 43 |
| 2015 | 1475 | 222 | 160 | 58 | 4 | 40 |
| 2016 | 1452 | 263 | 178 | 82 | 3 | 15 |
| 2017 | 1558 | 256 | 162 | 91 | 2 | 34 |
| 2018 | 1626 | 282 | 183 | 95 | 1 | 24 |
| 2019 | 1692 | 288 | 186 | 95 | 2 | 31 |
| 2020 | 1496 | 272 | 185 | 88 | 1 | 34 |
| 2021 | 1547 | 285 | 195 | 86 | 3 | 20 |
| 2022 | 1514 | 186 | 126 | 56 | 1 | 17 |
| All years | 14923 | 2325 | 1563 | 727 | 23 | 267 |
| *K. pneumoniae* |  |  |  |  |  |  |
| 2013 | 453 | 47 | 33 | 2 | 15 | 1 |
| 2014 | 512 | 59 | 37 | 4 | 21 | 1 |
| 2015 | 648 | 85 | 60 | 6 | 22 | 4 |
| 2016 | 671 | 96 | 79 | 8 | 14 | 5 |
| 2017 | 675 | 77 | 60 | 1 | 22 | 7 |
| 2018 | 688 | 93 | 70 | 5 | 20 | 2 |
| 2019 | 686 | 109 | 98 |  | 14 | 4 |
| 2020 | 684 | 121 | 100 | 5 | 18 | 4 |
| 2021 | 616 | 94 | 81 | 3 | 14 | 1 |
| 2022 | 629 | 93 | 82 | 4 | 9 | 3 |
| All years | 6262 | 874 | 700 | 38 | 169 | 32 |

Supplementary Table 2. Counts of Enterobacterales isolates

| **Organism/Year** | **No. of isolates** | | | | |
| --- | --- | --- | --- | --- | --- |
|  | **Total** | **CRE** | **Carbapenemase positive** | **KPC positive** | **NDM positive** |
| Enterobacterales |  |  |  |  |  |
| 2013 | 2690 | 86 | 79 | 79 |  |
| 2014 | 2737 | 57 | 40 | 39 | 1 |
| 2015 | 3284 | 81 | 55 | 53 | 3 |
| 2016 | 3342 | 51 | 48 | 46 |  |
| 2017 | 3488 | 41 | 37 | 35 |  |
| 2018 | 3706 | 37 | 31 | 25 | 4 |
| 2019 | 3725 | 28 | 21 | 16 | 1 |
| 2020 | 3580 | 29 | 25 | 22 | 2 |
| 2021 | 3653 | 64 | 52 | 39 | 7 |
| 2022 | 3496 | 35 | 24 | 19 | 3 |
| All years | 33701 | 509 | 412 | 373 | 21 |

Supplementary Figure 1. Activity of antimicrobial agents overtime tested against β-lactamase–producing isolates collected during 10 years in 22 US hospitals
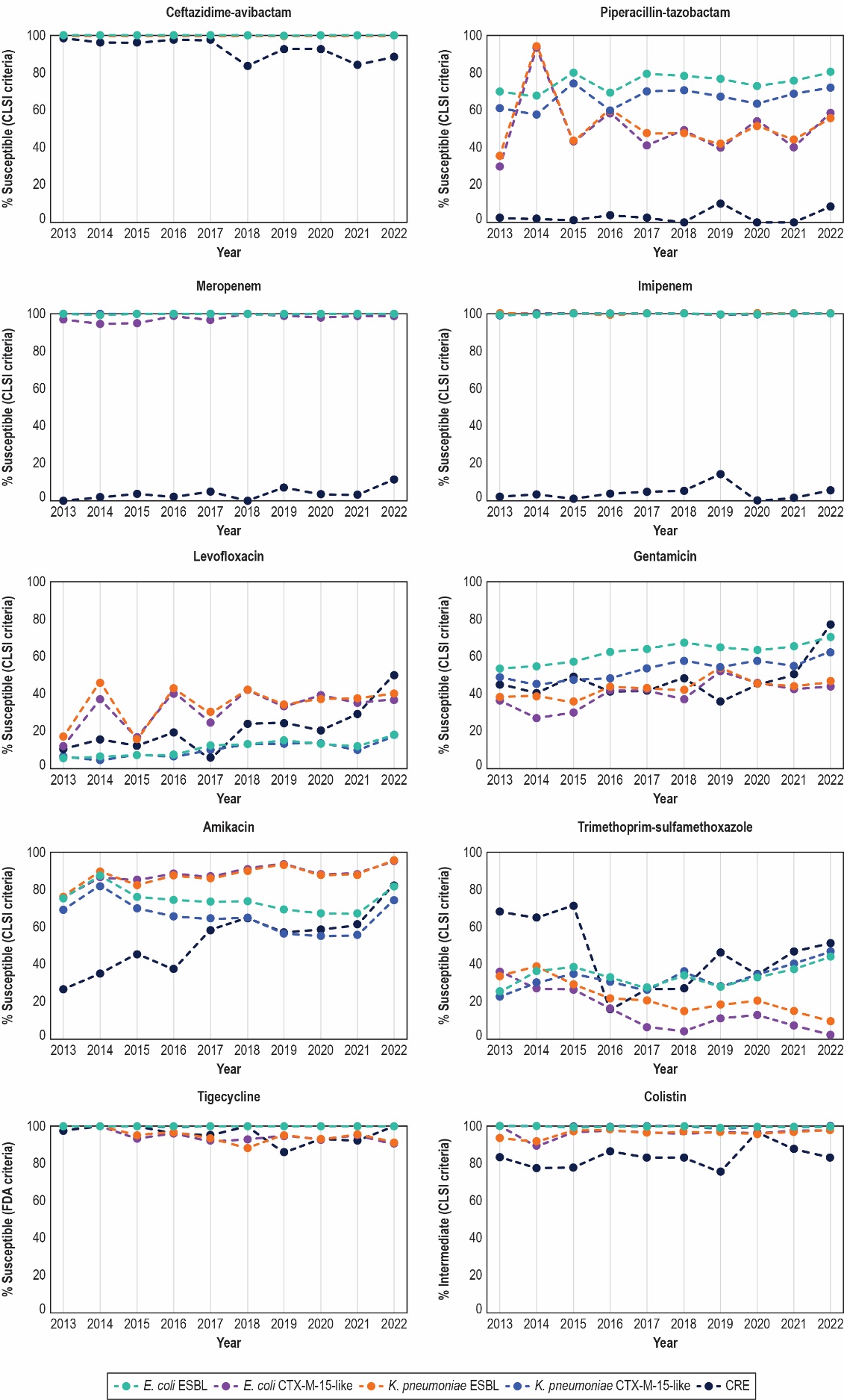

Supplement: dlag142_Supplementary_Data [file dlag142_supplementary_data.docx]
